# Molecular Dynamics of Chloroplast Membranes Isolated from Wild-Type Barley and a Brassinosteroid-Deficient Mutant Acclimated to Low and High Temperatures

**DOI:** 10.3390/biom11010027

**Published:** 2020-12-29

**Authors:** Iwona Sadura, Dariusz Latowski, Jana Oklestkova, Damian Gruszka, Marek Chyc, Anna Janeczko

**Affiliations:** 1 Polish Academy of Sciences, The Franciszek Górski Institute of Plant Physiology, Niezapominajek 21, 30-239 Kraków, Poland; ania@belanna.strefa.pl; 2 Department of Plant Physiology and Biochemistry, Faculty of Biochemistry, Biophysics and Biotechnology, Jagiellonian University, Gronostajowa 7, 30-387 Kraków, Poland; dariusz.latowski@uj.edu.pl; 3 Laboratory of Growth Regulators, The Czech Academy of Sciences, Institute of Experimental Botany & Palacký University, Šlechtitelů 27, 78371 Olomouc, Czech Republic; jana.oklestkova@upol.cz; 4 Institute of Biology, Biotechnology and Environmental Protection, Faculty of Natural Sciences, University of Silesia, Jagiellońska 28, 40-032 Katowice, Poland; damian.gruszka@us.edu.pl; 5 Department of Environmental Protection, Faculty of Mathematical and Natural Sciences, University of Applied Sciences in Tarnów, Mickiewicza 8, 33-100 Tarnów, Poland; mrsch7@gmail.com

**Keywords:** brassinosteroids, chloroplast membranes, temperature stress, barley, molecular dynamics, EPR

## Abstract

Plants have developed various acclimation strategies in order to counteract the negative effects of abiotic stresses (including temperature stress), and biological membranes are important elements in these strategies. Brassinosteroids (BR) are plant steroid hormones that regulate plant growth and development and modulate their reaction against many environmental stresses including temperature stress, but their role in modifying the properties of the biological membrane is poorly known. In this paper, we characterise the molecular dynamics of chloroplast membranes that had been isolated from wild-type and a BR-deficient barley mutant that had been acclimated to low and high temperatures in order to enrich the knowledge about the role of BR as regulators of the dynamics of the photosynthetic membranes. The molecular dynamics of the membranes was investigated using electron paramagnetic resonance (EPR) spectroscopy in both a hydrophilic and hydrophobic area of the membranes. The content of BR was determined, and other important membrane components that affect their molecular dynamics such as chlorophylls, carotenoids and fatty acids in these membranes were also determined. The chloroplast membranes of the BR-mutant had a higher degree of rigidification than the membranes of the wild type. In the hydrophilic area, the most visible differences were observed in plants that had been grown at 20 °C, whereas in the hydrophobic core, they were visible at both 20 and 5 °C. There were no differences in the molecular dynamics of the studied membranes in the chloroplast membranes that had been isolated from plants that had been grown at 27 °C. The role of BR in regulating the molecular dynamics of the photosynthetic membranes will be discussed against the background of an analysis of the photosynthetic pigments and fatty acid composition in the chloroplasts.

## 1. Introduction

Among the abiotic stresses, temperature stress is a global problem that mainly causes a decrease in the yield of most crop plants in agriculture and horticulture [1,2]. Some cereal species are sensitive to cold (maize), while other species (winter wheat) are sensitive to low temperatures, especially when there is poor snow cover on fields, which causes frost injuries that lower the yield. On the other hand, high temperatures may be particularly dangerous to plants when there is a water deficit in the summer vegetation season, which has increasingly happened in recent years due to climate changes. Periods of cold (e.g., 5 °C) harden plants (e.g., winter cereals) and enable them to better survive frost during winter. In turn, exposure to warmth may increase the ability of plants to survive high-temperature stress (e.g., 40 °C). Among the plant strategies for counteracting the negative effects of abiotic stresses (including extreme temperatures) [3], biological membranes are an important element. According to Horvath et al. [4], membranes can be considered to be “thermal sensors”, and are the primary cause of many other metabolic changes within cells and their organelles. During acclimation to changing temperature conditions, the alterations in the fluidity of the biological membranes are connected with changes in the proportion of unsaturated fatty acids, which results in a rearrangement of the membrane structure and its properties. The membrane properties can also be altered by incorporating various components into their structure, such as tocopherols, sterols, steroids [5], or carotenoids [6].

Many of the physiological phenomena that occur in cells could be due to the multidirectional effect of hormones on the plant metabolism, which is also the basis for the adaptability of organisms in order to function in changing environmental conditions. One plant hormone group is the brassinosteroids (BR), which are responsible for regulating both plant growth and development as well as their response to stress. BR belong to the steroid phytohormones that were first isolated from oilseed rape pollen in the 1970s [7,8]. To date, more than 70 BR are known, including brassinolide, 24-epibrassinolide, 28-homocastasterone, etc. Bajguz and Hayat [9] showed that in plants that had been exposed to temperature extremes, BR counteract the inhibition of growth, reduce the biomass losses and increase the plant survival rate. This is the result of the multidirectional activity of BR at the cellular and molecular level [10]. The function of BR in plants is still being explained and relatively little is known about their impact on the plant membrane properties. Recently, we found that BR may be involved in regulating the accumulation of the proteins that are incorporated in the cell membranes of barley plants (heat shock proteins, proton pumps and aquaporins) [11,12]. Our most recent studies revealed that barley mutants with a BR deficiency or a BR insensitivity were characterised by a different fatty acid composition in the cell membranes, which resulted in altered membrane physicochemical properties that could have an impact on the membrane-dependent physiological processes [13]. 

Studies of the compounds that are incorporated into the membranes have made a huge contribution to understanding the processes that occur in the biological membrane. In the case of thylakoids that had been obtained from the diatom *Phaeodactylum tricornutum*, studies of the diadinoxanthin cycle have provided information about the molecular dynamics of the thylakoid membrane and about the influence of the diadinoxanthin cycle pigment on this effect [14]. Strzałka et al. [15] investigated changes in the physical properties (general membrane lipid fluidity, dynamic orientational order parameter) of the vacuolar membranes compared to the protein body membrane in germinating pumpkin (*Cucurbita* sp.) seeds. Similar studies were performed on thylakoid membranes that had been isolated from barley during leaf senescence [16]. These measurements are taken using electron paramagnetic resonance (EPR) spectroscopy, which permits the direct study of the systems (including biological systems) that contain unpaired electrons. In biological systems, compounds that contain unpaired electrons are present in the products of biochemical transformations, e.g., photosynthesis. EPR can also be used to study systems that do not contain unpaired electrons naturally. In this case, artificial paramagnetic centres (spin labels) that contain a nitroxyl group, e.g., 5-doxyl stearic acid (5-SASL) or 16-doxyl stearic acid (16-SASL) are incorporated into the studied object. In the case of the biological membranes, the EPR spectra can provide information, among others, about the degree of order (S) and the rotational correlation times (τ_B_ and τ_C_) of the molecules that contain a spin label, which is helpful in determining the molecular dynamics of the membranes [14,15]. 

In the presented study, we focused on the chloroplast membranes, which are crucial elements of photosynthetic machinery, and affect the level and quality of plant yields [17]. Bearing in mind the role of membranes as the first cellular line to react to changing temperatures (and also the importance of chloroplasts for the crucial plant process of photosynthesis), the aim of the study was to broaden the knowledge about the changes in the molecular dynamics of the chloroplast membranes during the acclimation of a plant to extreme temperatures and the influence of BR on these properties. By studying wild-type barley plants and a BR-deficient mutant, we wanted to answer the following detailed questions: (1) is the content of BR in the chloroplast membranes different in the wild-type plants than in a BR-biosynthesis mutant grown at an optimal growth temperature, i.e., 20 °C? (2) How does the temperature of acclimation (5 °C and 27 °C) modify the accumulation of BR in the chloroplast membranes of the wild type and mutant? (3) Are there differences in the molecular dynamics of the chloroplast membranes from wild-type barley and a mutant that is cultured at 20 °C? (4) How does a lower (5 °C) or higher (27 °C) temperature during plant growth modify the molecular dynamics of the chloroplast membranes? Finally, the role of BR as regulators of the molecular dynamics of the photosynthetic membranes will be discussed in light of the chloroplast content of the photosynthetic pigments and fatty acid composition in the BR-deficient barley mutant and in the respective wild type. 

## 2. Material and Methods

### 2.1. Plant Material, Experimental Design and Sampling

The objects of study were the barley cultivar Delisa (wild type) and its semi-dwarf mutant (522DK), which has disturbances in the BR biosynthesis. The mutant was described in the work by Gruszka et al. [18]. The mutant was obtained via the chemical mutagenesis of the cultivar Delisa. The 522DK mutant has a G > A substitution at position 1130 of the *HvDWARF* transcript [18] and at position 3031 in the gene sequence [19], which causes the change of the valine-341 residue into isoleucine. The substituted valine-341 is a highly conserved residue that occurs in similar position in the homologous DWARF polypeptides from barley, *Arabidopsis*, rice and tomato. Since the *HvDWARF* gene encodes the brassinosteroid C6-oxidase, which is an important factor in the BR-biosynthesis (it catalyses the production of castasterone), the mutant 522DK has a lowered content of castasterone [19] and it also has a decreased level of other BR [20].

The seeds were germinated for three days at 24 °C in the dark. After germination, the seedlings were transplanted into pots (40 cm × 15 cm × 15 cm) (about 40 seedlings per pot) containing soil (‘Eco-ziem universal soil’ (Jurków, Poland), soil from the cultivation plots at the University of Agriculture (Cracow, Poland), sand and ‘Substral Osmocote—a universal substrate’ (Scotts Poland sp. z o.o., Warsaw, Poland) at a ratio of 8:4:2:4, respectively), after which they were grown for 18 days at 20 °C (16 h photoperiod). Then, the plants were divided into two groups. Each group consisted of three pots with plants of the wild-type cultivar and three pots with plants of the mutant. In the first group, the temperature was lowered to 5 °C (21 days, 8 h photoperiod) and in the second group, it was increased to 27 °C (7 days, 16 h photoperiod). Light intensity in the growth chambers was 170 μmol m^−2^ s^−1^ and was provided by HPS Philips SON-T AGRO 400 W lamps. Because the current studies are a continuation of our earlier works, the durations of the acclimation and the photoperiods were identical to those that were described in our earlier articles [11,12,20,21,22]. According to literature data, both cold acclimation and acclimation to higher temperatures enable plants to acquire a greater tolerance to frost or heat, respectively [23,24,25]. Our previous studies [20] showed that after acclimation at 5 °C, both the wild type and mutant were characterised by a comparable tolerance to frost (−8 °C) while the mutant 522DK had an even better frost tolerance at −6 °C compared to the wild-type Delisa. After acclimation at 27 °C, the mutant had a much better heat tolerance (tested at 38 and 45 °C) compared to the wild type. 

Before cutting off the plants, leaf greenness was measured using a chlorophyll meter for the plants that had been grown at 20 °C and then in the plants that had been acclimated at 5 °C and 27 °C. The plants grown at 20 °C had four leaves, while the acclimated plants had four well-developed leaves and sometimes a young fifth leaf. The samples to isolate the chloroplasts were collected from the wild-type and mutant plants that had been grown at 20 °C and from the plants that had been acclimated at 5 °C and 27 °C. It was possible to isolate about 1.5 g (0.3 g per sample) of chloroplasts from 20 to 25 plants that weighed around 40 g. Three samples containing 0.3 g of chloroplasts (each sample from plants from different pot) were collected to analyse the BR. For the other analyses, each sample containing 0.3 g chloroplasts were first suspended in a 1 mL chloroplast isolation buffer (CIB) buffer (see *Isolation of Chloroplasts* section) and these suspensions were used to further analyse the content of proteins, photosynthetic pigments, and the fatty acid composition. The proteins were also analysed in order to standardise the samples to be used for the EPR studies.

### 2.2. Leaf Greenness Measurement

Leaf greenness, which corresponds to the leaf chlorophyll concentration, was measured using a chlorophyll meter (SPAD 502; Konica Minolta, Tokyo, Japan, (SPAD—Soil Plant Analysis Development)) and the values of greenness are expressed in arbitrary SPAD units. The measurement was taken in the middle part of the fourth fully developed leaf in three technical replications per each leaf and the average value was calculated for each leaf. The leaves from nine plants were measured—three plants from each pot. 

### 2.3. Isolation of Chloroplasts from Barley Leaves

The chloroplasts were isolated based on a modified protocol of Block et al. [26] and Filek et al. [27]. About 100 g of the aerial part of the barley (mainly leaves) were homogenised using a Camry CE 4050 blender in 400 mL of a chloroplast isolation buffer (CIB) (pH 7.5) that contained 50 mM Tris-HCl, 5 mM ethylenediaminetetraacetic acid (EDTA) and 0.33 mM sorbitol. The crude extract was filtered and centrifuged for three minutes at 300× *g* (Hettich zentrifugen Universal 320R, rotor 1494, Tuttlingen, Germany) in order to remove any residues after plant homogenisation. Next, the supernatant was centrifuged for ten minutes at 1200× *g* (Hettich zentrifugen Universal 320R, rotor 1494, Tuttlingen, Germany). The obtained pellet contained isolated chloroplasts. The purity of the isolated chloroplasts was checked under a microscope (Nikon Eclipse E600, Tokyo, Japan) (Figure S1). The entire isolation process was performed in a cold room (4–6 °C). Next, in order to break down the chloroplasts, samples were frozen in liquid nitrogen and thawed in two cycles; this was especially important in EPR studies. Breakdown of chloroplasts was checked under microscope (Nikon Eclipse E600, Tokyo, Japan). Frozen samples were kept at −80 °C until the day of analysis. 

### 2.4. Analysis of the Protein Content in Chloroplasts

The protein concentration was estimated according to Sedmak and Grossberg [28]. Two microlitres of a 10% water solution of Triton X-100 (Sigma-Aldrich, Poznań, Poland) were mixed with 2 μL of the chloroplasts that had been suspended in CIB and 196 μL of a CIB buffer (see *Isolation of chloroplasts* section) and kept for 15 min on ice. Ten microlitres of each sample were placed into separate wells of a plate (96-well polystyrene titration plate with a flat bottom, FL Medical, Torreglia, Italy) and then, 200 μL of a Bradford reagent (BioRad, Munich, Germany) (diluted with water 1:4) was added. After 10 min, the absorbance was recorded (595 nm) using a Synergy^TM^2 Multi-Detection Microplate Reader (BioTek, Winooski, VT, USA). The measurements were carried out in three replicates. Bovine serum albumin (BSA) (Sigma-Aldrich, Poznań, Poland) was used as the calibration standard. The BSA for spectrophotometric measurements was diluted in the same buffer as the buffer that had been used to isolate the chloroplasts (CIB buffer).

### 2.5. Analysis of the Fatty Acid Composition in Chloroplasts

The fatty acids (FA) were extracted by homogenising the isolated chloroplasts. Samples of the chloroplast suspension in the CIB buffer (0.15 mL each sample) were suspended in 0.20 mL of toluene and transferred into screw-capped glass test tubes. Subsequently, 1.50 mL of methanol and 0.30 mL of an 8.0% HCl solution were added. The test tubes were vortexed and then incubated at 45 °C overnight in order to turn the extracted fatty acids into fatty acid methyl esters (FAME). After cooling to room temperature, the FAME were extracted by adding 1 mL of hexane and 1 mL of water [29]. The test tubes were vortexed, and then the hexane layers were analysed using gas-liquid chromatography (GC). The n-hexane extracts were analysed chromatographically on an Agilent 6890N gas chromatograph (Agilent Technologies, Santa Clara, CA, USA) that was equipped with a flame ionisation detector (FID) and a capillary column. The certified reference material 37 FAME MIX (Supelco, CRM 47885) and an internal standard (biphenyl) were used to identify and quantify the fatty acid profiles. The analyses were conducted using a gas chromatograph equipped with a split/splitless injector. An ionic liquid fused silica capillary column (SLB-IL100, Supelco, 28884-U) (30 m × 0.25 mm ID × 0.2 mm film thickness) with a matrix 1,9-di(3-vinylimidazolium)nonane bis(trifluoromethanesulfonyl)imide phase was operated under the following programmed conditions: 50–240 °C at 3 °C min^−1^ for 30 min (detector and injector temperatures of 240 °C), autosampler injection mode and volume of 0.5 μL and split (10:1) with helium 6.0 as the carrier gas (velocity 40 cm min^−1^). FAME were identified by comparing them with the standard mixture (Sigma-Aldrich, Poznań, Poland) and their retention time.

### 2.6. Determining the Carotenoid and Chlorophyll Content in Chloroplasts

Eighty percent aqueous acetone (2.09 mL) was added to the samples of the suspension of chloroplasts in the CIB buffer (0.01 mL each sample). The samples were vortexed and centrifuged (8 min, 12.100× *g*) (Eppendorf Mini-Spin, Hamburg, Germany). The UV-ViS (Jasco870, Easton, MD, USA) measurements were taken at wavelengths of 663.2, 664.8 and 470 nm. The measurements were taken in three replicates. The amounts of chlorophylls and carotenoids were calculated according to following Equations [30]:

C_a_ = 12.25·A663.2 − 2.79·A664.8 (1)

C_b_ = 21.50·A664.8 − 5.10·A663.2 (2)

C_a+b_ = 7.15·A663.2 + 18.71·A664.8 (3)

C_x+c_ = (1000·A470 − 1.82·C_a_ − 85.02·C_b_)/198 (4)

where C_a_ is the concentration of chlorophyll *a*; C_b_ is the concentration of chlorophyll *b*; C_a+b_ is the sum of the chlorophyll *a* and *b* concentration; and C_x+c_ is the concentration of carotenoids.

### 2.7. Analysis of the Brassinosteroids in the Chloroplasts

Samples of the isolated chloroplasts (0.3 g each) were homogenised in 80% methanol and enriched to the internal standard with deuterium-labelled BR (25 pmol/sample, Olchemim s.r.o., Olomouc, Czech Republic). Next, the samples were centrifuged and the supernatant was passed through Discovery DPA-6S columns (Supelco, Bellefonte, PA, USA) and immunoaffinity columns (Laboratory of Growth Regulation, Olomouc, Czech Republic) [31]. The brassinosteroids that had been eluted with cold 100% methanol from the IA columns were dried and resuspended in 40 μL of methanol in order to measure them on a UHPLC using a tandem mass spectrometry (UHPLC–MS/MS) with an ACQUITY UPLC^®^ I-Class System (Waters, Milford, MA, USA) and a Xevo™ TQ-S MS triple quadrupole mass spectrometer (Waters MS Technologies, Manchester, UK) [31,32]. The analyses were performed in three repetitions and each repetition included about 0.3 g of the isolated chloroplasts.

### 2.8. Electron Paramagnetic Resonance (EPR) Measurements 

**Figure 1:**
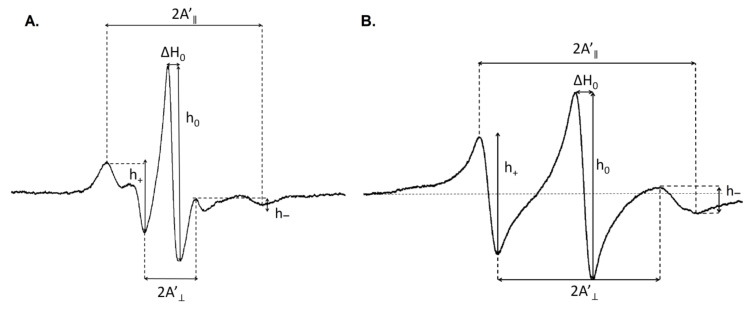
Schematic electron paramagnetic resonance (EPR) spectra of the SASL-5 (**A**) and SASL-16 (**B**) spin labels that had been incorporated into the chloroplast membranes. The spectral parameters that were analysed are indicated (2A’_⊥_ and 2A’_||_, ΔH_0_, and h_+_ , h_0_ and h_−_).

The molecular dynamics of the cell membranes were determined according to the modified protocol described by Strzałka et al. [15] and was monitored using EPR spectroscopy with spin labels 5-doxyl stearic acid (5-SASL, Santa Cruz Biotechnology, Heidelberg, Germany) and 16-doxylstearic acid (16-SASL, Sigma-Aldrich, Poznań, Poland), which provides information about the molecular dynamics of the region near the polar heads and the membrane interior, respectively. The spin labels were incorporated into the chloroplast membranes at room temperature by vortexing. About 100–300 μL of the chloroplast membranes (corresponding to 30 μg of protein) were vortexed for 20 min with 5 μL of 10 mM spin labels in methanol [15]. To remove any traces of the spin labels in the supernatant, the samples were centrifuged three times at 4500× *g* for two minutes at room temperature. The pellet that was obtained was resuspended in 50 μL of a 10mM CIB buffer (its composition is described in the *Isolation of chloroplasts* section) in order to obtain a final cell membrane concentration yield that corresponded to 30 μg of the protein (the protein content was measured using the Bradford method and no significant differences were observed between wild-type Delisa and the 522DK mutant). The EPR spectra of the spin label as a function of the temperature was recorded using an X band EPR spectrometer (Miniscope, Berlin, Germany) that was equipped with a Temperature Controller (Magnettech, Berlin, Germany) with a temperature range of 0 to 40 °C at intervals of 5 °C. For the plants that had been acclimated at 5 °C, the range of the measurement was between 0 °C and 25 °C and the interval was 2.5 °C. The EPR measurements were performed at microwave powers between 3.2 mW and 10.0 mW with a sweep width of 12–15 mT, a modulation amplitude of 0.1 mT and a microwave frequency of about 9.4 GHz. Two biological replicates with two to three technical replicates were done. The obtained EPR spectra were analysed with MultiPlot 2.0 Software (Magnettech GmBH, Berlin, Germany) (Figure 1).

The parameters that were required to calculate the dynamic orientational order parameter (S) and the rotational correlation times (τ_2B_ and τ_2C_) are presented in Figure 1. The values of the S parameter were calculated using the following Equation [33]:

where S = 0.5407 (A’_||_ − A’_⊥_)/a_o_ (5)

a_o_ = (A’_||_ + 2A’_⊥_)/3 (6)

The rotational correlation times (τ_2B_ and τ_2C_) were calculated according to the following Equations [34]:

τ_2B_ = 6.51 · 10^−10^ · ΔH_0_ · ((h_0_/h_−_ )^½^ − (h_0_/h_+_ )^½^ ) [s] (7)

and

τ_2C_ = 6.51 · 10^−10^ · ΔH_0_ · ((h_0_/h_−_ )^½^ + (h_0_/h_+_ )^½^ − 2) [s] (8)

### 2.9. Statistical Analysis

**Figure 2:**
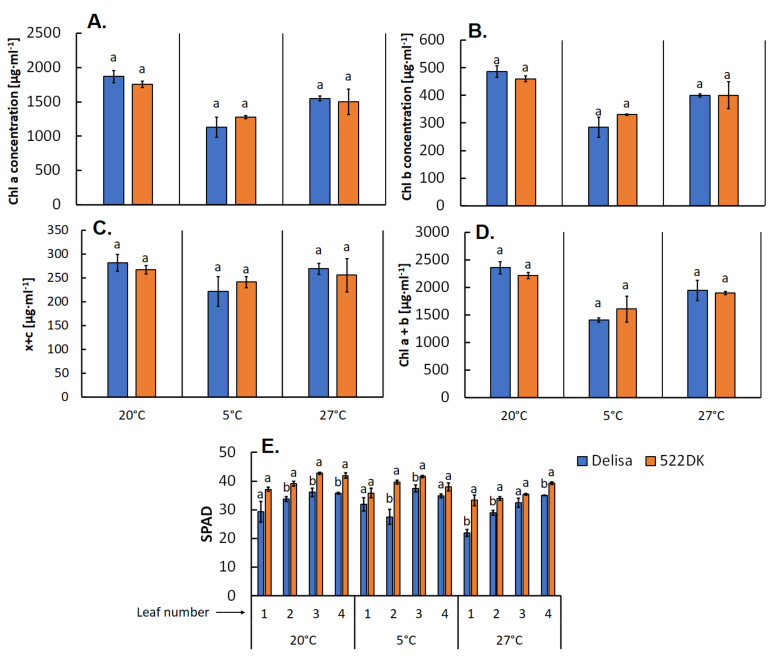
Concentration of chlorophyll (Chl) *a* (**A**), chlorophyll *b* (**B**), xantophylls and carotenoids (x + c) (**C**), and total chlorophyll (*a* + *b*) (**D**) in a suspension of chloroplasts from the barley wild-type Delisa and its 522DK mutant. (**E**) The greenness intensity [SPAD units] of 1st–4th leaf of Delisa and its mutant 522DK. Plants had been cultivated at 20 °C and acclimated at 5 °C and 27 °C. Mean values (±SE) marked with the same letters (separately for plant growth temperature) did not significantly differ according to Student’s *t*-test (*p* ≤ 0.05).

**Figure 3:**
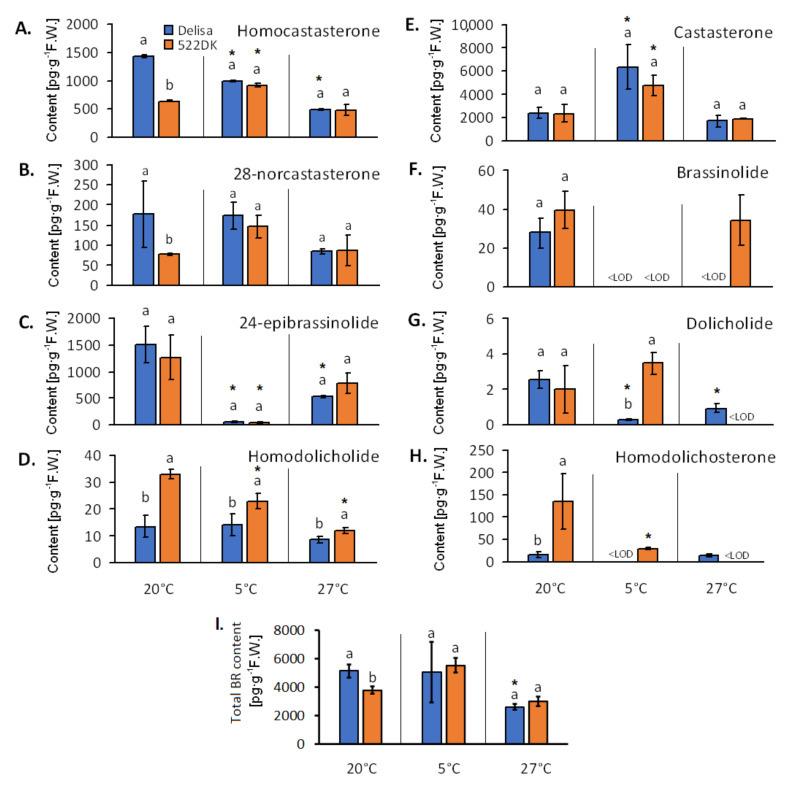
Content of brassinosteroids in the chloroplasts of the barley wild-type Delisa and the BR-deficient mutant 522DK that had been grown at 20 °C and acclimated at 5 °C or 27 °C. (**A**) Homocastasterone, (**B**) 28-norcastasterone, (**C**) 24–epibrassinolide, (**D**) Homodolicholide, (**E**) Castasterone, (**F**) Brassinolide, (**G**) Dolicholide, (**H**) Homodolichosterone, (**I**) Total BR content. <LOD–below detection limit. Mean values (±SD) marked with the same letters are not significantly different according to Student’s t-test (*p* ≤ 0.05) (separately for each growth temperature). Additionally, in order to show the impact of temperature on the BR level in Delisa and mutant, the comparisons (Student’s *t*-test, *p* ≤ 0.05) were performed in pairs (Delisa at 20 °C and 5 °C; mutant at 20 °C and 5 °C; Delisa at 20 °C and 27 °C; mutant at 20 °C and 27 °C) and any significant differences are indicated by an “*”.

**Figure 4:**
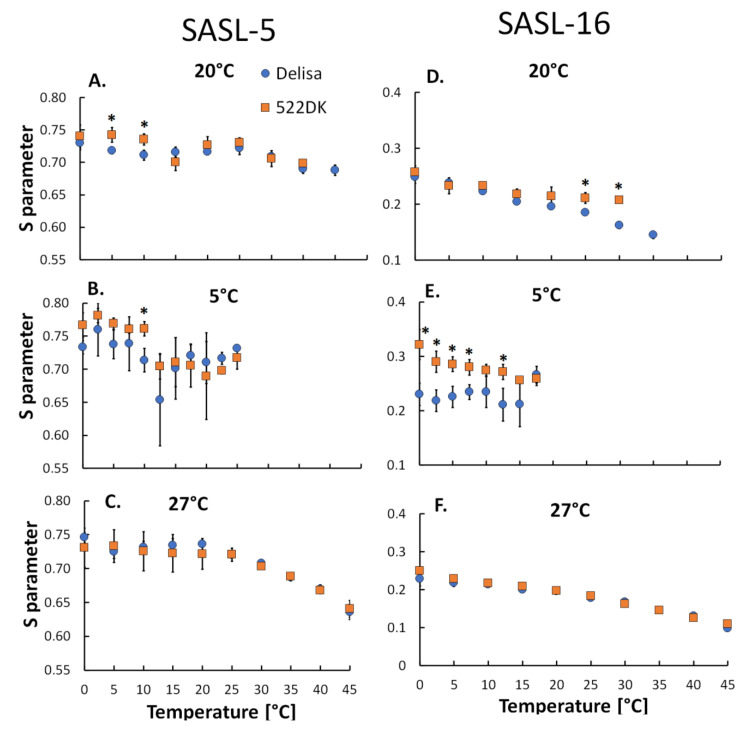
Dependence of the dynamic orientational order parameter S that was calculated for the chloroplast membranes that had been isolated from the barley wild-type Delisa and its mutant 522DK with incorporated SASL-5 (**A**–**C**) and SASL-16 (**D**–**F**) on temperature. Plants had been cultivated at 20 °C and acclimated at 5 °C and 27 °C. Average data are given ± SE and significant differences between the wild type and mutant are indicated with an asterisk (*); comparisons were made separately for each EPR measurement temperature.

The statistical analysis (ANOVA, post hoc test) was performed using Statistica 13.1 (StatSoft, Tulsa, OK, USA). For the statistical analysis in the study, when the Delisa and 522DK were compared, Student’s t-test was used (*p ≤* 0.05). Values marked with the same letters in Figure 2 and Figure 3 and Table 1 did not differ significantly (the comparisons were performed separately for each temperature of plant growth). Moreover, the accumulations of BR (Figure 3) in the Delisa cultivar and mutant at different temperatures were also compared (Student’s t-test, *p ≤* 0.05). The comparisons were performed in pairs (Delisa for 20 °C and 5 °C; mutant for 20 °C and 5 °C; Delisa for 20 °C and 27 °C; mutant for 20 °C and 27 °C) and any significant differences are indicated by an “*”. The significant differences in the results for the molecular dynamics (Figure 4) between the wild-type Delisa and the mutant are indicated by an asterisk (*); the comparisons were performed separately for each EPR measurement temperature. 

## 3. Results and Discussion

### 3.1. Characteristic of Selected Chemical Components of the Chloroplast Membranes Isolated from Barley Grown at 20 °C, 5 °C, and 27 °C

#### 3.1.1. Fatty Acid Composition of the Chloroplast Membranes

Only a few FA had differences in content between the wild-type Delisa and its 522DK mutant and even when they were statistically significant, they were not huge. Those that were most abundant in the chloroplast membranes were 18:3 (about 60%) and 16:0 (about 16–18%). The content of the other FA, including 18:1^Δ9cis^ or 18:2^Δ6cis^, was less than a few percent. What is important is that at all of the tested temperatures, there were no differences between the mutant and Delisa for the main FA (18:3). As for the other FA, for example, at 20 °C the 522DK mutant was characterised by a higher content of 16:0 and 18:1^Δ9cis^ than the Delisa cultivar, while at 5 °C, only 18:1^Δ9cis^ content was higher in the 522DK mutant. For the plants that had been acclimated to a high temperature, the 522DK mutant was characterised by a higher level of 18:2^Δ6cis^ than the Delisa cultivar (Table 1). 

There were no significant differences between Delisa and its mutant at any growth temperature for the 18:3/18:2 ratio. Only at 20 °C was there a significantly higher value of the unsaturated FA to saturated FA ratio (U/S) for Delisa than for the 522DK mutant (Table 1).

Fatty acids are the main component of the biological membranes and their composition plays a huge role in the membrane properties including membrane fluidity [35,36]. According to Hölzl and Dörmann [37], chloroplasts are the major sites for FA synthesis in plant cells and 16:0, 18:1 ^Δ9cis^, 18:2 ^Δ9cis,12cis^ and 18:3 ^Δ9cis,12cis,15cis^ FA are present at the highest levels in the chloroplast membranes. Therefore, our data are generally consistent with this [37] and other previous works on chloroplasts that had been isolated from avocado fruits and cauliflower leaves [38], as well as on the callus of winter oilseed rape [39] and on the leaves of *Pisum sativum* L, winter wheat, and barley [13,40,41]. 

#### 3.1.2. Carotenoid and Chlorophyll Content in the Chloroplast Membranes 

Analysis of chlorophyll *a*, *b* and total chlorophylls (*a* + *b*) in the isolated chloroplasts showed no differences in the content of these pigments between the wild type and the mutant (Figure 2A,B,D). However, the leaf greenness measurements that were taken using a noninvasive method (chlorophyll meter) showed that for all four of the analysed leaves at all growth temperatures, the mutant was characterised by a more intense greenness (Figure 2E). Moreover, interestingly, according to our previous studies [42], a spectrophotochemical analysis of the chlorophyll content (especially chlorophyll *b*) in the leaf dry matter showed that 522DK had a lower content of chlorophyll than the Delisa cultivar, which was consistent with numerous studies that have proven that BR are important regulators of the chlorophyll accumulation because exogenous BR usually have caused an increase in chlorophyll content especially under stressful conditions [10]. In our current studies, the “greener” leaves of the 522DK mutant was rather a result of the mutant’s semi-dwarfness (more compact cells with a higher density of chloroplasts/chlorophyll per leaf surface), but again an analysis of the pure chloroplast suspension revealed no difference between the wild type and the mutant. In this experiment, the content of chlorophyll in the chloroplasts was measured because these pigments also play an important role in membrane fluidity [6,43]. Because the chlorophyll contents in the chloroplast membranes of 522DK and Delisa were similar, we can assume that the chlorophylls regulated the fluidity of the chloroplast membranes in comparable manner. 

Similar to chlorophyll, the contents of xanthophylls and carotenoids in the pure chloroplasts were also at the same level in the wild type and the mutant (Figure 2C). Interestingly, our earlier studies [42] showed that the BR-deficient mutant 522DK was characterised by a higher accumulation of carotenoids than Delisa when calculated per leaf dry mass. Contrary to these findings, Nie et al. [44] reported that carotenoid level increased in *Solanum lycopersicum*, which had a higher endogenous level of BR compared to its wild type. On the other hand, Koĉova et al. [45] found that exogenous brassinosteroids did not significantly affect the carotenoid content in maize leaves. Although the role of BR in the accumulation/biosynthesis of carotenoids seems to be ambiguous, it is certain that carotenoids have huge influence on the thylakoid membrane fluidity and they are also thought to be responsible for reducing the membrane fluidity of a membrane [6,43]. Because no differences in the carotenoid content in the chloroplasts of the mutant and wild type were observed in this study, we suspect that in the current experiment, the influence of carotenoids on the chloroplast membrane fluidity was comparable for Delisa and its mutant.

#### 3.1.3. Brassinosteroid Content of the Chloroplasts

In the chloroplasts, we were able to determine the following BR: brassinolide, castasterone, 28-homocastasterone, 24-epibrassinolide, 28-norcastasterone, dolicholide, homodolicholide and homodolichosterone (Figure 3A–H). 

Compared to the wild type, the BR-deficient mutant 522DK that had been cultured at 20 °C was characterised by a lower content of homocastasterone and 28-norcastasterone but higher content of homodolicholide and homodolichosterone in its chloroplasts. The other BR were at comparable level in the chloroplasts of both genotypes at this temperature. 

In the mutant that had been grown at 5 °C, there was a higher content of homodolicholide, homodolichosterone and also dolicholide compared to the wild type (Figure 3D,G,H). The homodolichosterone level was even below detection limit in the wild type at 5 °C (Figure 3H). As for the other BR at 5 °C, there were no significant differences between the mutant and its wild type. Contrary to 20 °C, brassinolide was not detected at 5 °C in either the mutant or the wild type (Figure 3F). 

As for the plants that had been acclimated at 27 °C, brassinolide was only detected in the chloroplasts of the mutant but not in the wild type (Figure 3F). While the chloroplasts of the 522DK plants at this temperature were characterised by a higher content of homodolicholide than the wild type, the level of dolicholide and homodolichosterone was below the detection limit in the chloroplasts of the mutant (Figure 3D,G,H). The other BR had a comparable concentration in the chloroplasts of both genotypes at this temperature. 

As for the total amount of the detected BR (sum of all of the detected BR) (Figure 3I), the obtained results confirmed that the 522DK mutant was generally BR-deficient, but this was only observed at 20 °C for the chloroplasts. Interestingly, our earlier analysis of the BR content in the leaves of Delisa and 522DK [20] unambiguously showed that the mutants accumulated less BR in their leaf tissue than the wild type at all three temperatures (20 °C, 5 °C and 27 °C). Moreover, in the leaf material, only brassinolide, castasterone, and homocastasterone were found in that study, while in this study, many more BR were detected in the pure chloroplasts, including dolicholide, dolichosterone, homodolicholide, and also 28-norcastasterone and 24-epibrassinolide. Interestingly, dolicholide had already been detected by us in the barley leaves of other cultivars [46], but not in Delisa or its 522DK mutant [20]. In our opinion, the differences in the detectable BR profile in the Delisa and 522DK leaves and chloroplasts are due to the fact that in the case of the leaves, 300 mg of fresh leaf material was used for the BR extraction, while in the case of the chloroplast isolation, 40 g of the leaves (25 plants) were used to obtain 1.5 g of the chloroplasts (300 mg pure chloroplasts per sample for the BR analysis). For this reason, the presence of BR that were revealed in the chloroplast samples here was probably below the detection limit in the whole leaves in earlier studies. 

The detection of BR in the barley chloroplasts confirms our earlier reports (research on wheat [27]) that these compounds accumulate in the chloroplasts. Based on the relatively limited knowledge of BR synthesis/decomposition sites in a cell, it is assumed that all BR biosynthetic enzymes belong to the family Cyt P450s and are located in the reticulum membrane [47]. The fact that BR accumulates in the chloroplasts (probably also in the membranes) of both the 522DK mutant and its wild type suggests that these hormones could be very important for the appropriate functioning of the chloroplasts. The phenomenon of the incorporation of BR in the chloroplast structure will require further research, even though some guidance regarding the role of BR was provided by Cai et al. [48], Zhang et al. [49] and Krumova et al. [50]. Exogenous BR prevented injuries to the chloroplast ultrastructure in plants that had been exposed to high and low temperatures [48,49]. However, according to Krumova et. al. [50], BR have an impact on the functioning and regulation of the photosynthetic apparatus and the architecture of the thylakoid membrane. The authors found that *Arabidopsis* BR mutants, which overexpress the BR receptor and are deficient in the BR biosynthesis, had increased thylakoid area compared to the wild type, which could be connected with changes in the chloroplast division mechanisms. On the other hand, at 20 °C, 5 °C, and 27 °C, the 522DK mutant had a better (than the wild type) efficiency of PSII (photosystem located in chloroplasts) [20]. Simultaneously (according to the current study), the mutant unambiguously accumulated only one BR—homodolicholide—in the chloroplasts at a higher amount than the wild type at all of the temperatures. Homodolicholide is a lesser-known BR and is present in relatively small amounts compared to castasterone or homocastasterone (Figure 3A,D,E). Might it then be responsible for the better performance of the chloroplasts in the mutant that had been exposed to temperature stress? This will also require further studies. 

As was mentioned above, the leaves of the mutant always had less BR than the leaves of the wild type [20]. In the case of mutant chloroplasts, however, we did observe: (1) the occurrence of lower amounts of BR, e.g., homocastasterone at 20 °C than in the wild type; (2) no differences in the BR content in the mutant and in the wild type (castasterone at all of the growth temperatures) and (3) a higher amount of BR in the mutant, (e.g., homodolicholide, independent of growth temperature). 

In our opinion, this fact suggests that the presence of BR in the chloroplasts and perhaps their incorporation into the membranes is driven by accumulation mechanisms that do not have much in common with their biosynthesis process (and final BR production). Perhaps, the purely physicochemical mechanisms are involved (the membrane lipid environment was “beneficial” for the accumulation of specific steroids—especially homodolicholide in the mutant). Temperature also had an effect on the accumulation of BR in the chloroplasts. Moreover, as was mentioned above, previous studies have shown that the presence of mycotoxins, the application of exogenous brassinosteroids in a culture medium or the cultivar’s tolerance to stress all influenced/modified the BR concentration in the chloroplasts in a different manner. A summary of the changes in the BR content in the chloroplast membranes of 522DK at different temperatures compared to its wild type is presented in Table S1.

### 3.2. Molecular Dynamics of the Barley Chloroplasts Measured Using EPR Spectroscopy

To study the molecular dynamics of the chloroplasts that had been isolated from the barley plants, two kinds of spin labels were incorporated into the membranes: SASL-5 and SASL-16. The first has a nitroxide group that is bound to the 5th carbon of the stearic acid and that is why it can provide information about the molecular dynamics processes that occur near the membrane surface. SASL-16 has a nitroxide group that is attached to the 16th carbon of the stearic acid and its incorporation into membranes provides information about the dynamic processes that occur inside the membrane [15]. The results of our experiment are presented in Figure 4A–F. It was found that the possibility to measure the spectrum of the spin labels (SASL-5 and SASL-16) was dependent on the ambient temperature of the plant from which the chloroplasts had been isolated. For example, for the chloroplasts that were isolated from the plants acclimated at 5 °C with incorporated SASL-16, it was possible to calculate the S parameter in a measurement of the temperature in the range of 0–17.5 °C (Figure 4E). On the other hand, for the chloroplasts that were isolated from the plants acclimated at 27 °C with incorporated SASL-16, it was possible to calculate the S parameter in the entire measurement temperature range that was used in this study, i.e., 0–45 °C. This shows that plant acclimation at 5 °C and 27 °C resulted in a good adaptation of the studied barley plants to low and high temperatures, respectively. Moreover, the dependence of the dynamic orientational order parameter on the temperature at which the plants were cultivated was also observed (Figure S2). As was mentioned earlier, according to our previous work [20], after acclimation at 5 °C and 27 °C, the 522DK mutant and its wild-type Delisa developed a tolerance to frost (up to −8 °C) and heat (38 °C and 45 °C). Additionally, in the study of Bojko et al. [51] on thylakoid membranes of *T. pseudonana* that were cultured at low and high temperatures (12 °C and 20 °C, respectively), a similar effect of efficient adaptation to acclimating temperatures was observed, which was connected, among others, with the molecular dynamics results. 

The dynamic orientational order parameter (S) that was calculated from SASL-5 and SASL-16 spectra gradually decreased for the chloroplast membranes from both the wild-type Delisa and the mutant along with an increase in the measurement temperature (Figure 4; Figure S2). This effect was connected with an increase in the molecular dynamics of the membranes and a less ordered molecular environment of the spin labels during an increase in the measurement temperature [14]. The obtained values of S are in agreement with the literature in which the dynamic orientational order parameter S varies between 1 (maximally ordered membranes) and 0 (maximally disordered membranes) [15,52]. An increase in the molecular dynamics is considered to represent an increase in membrane fluidity [53]. 

For the chloroplast membranes with an incorporated SASL-5 spin label in the measurement temperature range between 5 °C and 10 °C, the 522DK chloroplast membranes from the mutant that had been cultured at 20 °C were characterised by higher values of the S parameter than the Delisa’s membranes (Figure 4A). There were no significant differences between the chloroplast membranes of Delisa and 522DK for the other measurement temperatures (Figure 4A). For the chloroplast membranes of the plants that had been acclimated at 5 °C and 27 °C, there were generally no noticeable differences between the wild-type Delisa and the mutant (Figure 4B,C); the only exception was at a measurement temperature of 10 °C, when the membranes of the mutant were characterised by a higher value of S (Figure 4B). To summarise, near the membrane surface, there were no differences between the Delisa cultivar and its mutant 522DK that had been acclimated at 5 °C and 27 °C. This is consistent with the fact that the surface area of membranes needs to be quickly ordered in order to support the proper interactions of the membranes with their neighbour area and it is also needed in order to avoid the uncontrolled diffusion of molecules such as oxygen or free radicals, which can cause destructive reactions inside membranes that are not fully adapted to the new conditions of environmental stimuli [14,52,54].

For the chloroplast membranes with an incorporated SASL-16 spin label, differences in the S parameter were observed between the mutant and the wild type that had been acclimated at 5 °C. The values of S parameter were significantly lower for the Delisa cultivar compared to its mutant in the entire tested temperature range and the differences were almost always statistically significant (Figure 4E). The same effect was observed for the membranes of the plants that were cultured at 20 °C, but only in the measurement temperature range between 25 °C and 30 °C (Figure 4D). In this case, interestingly, the S parameters were measurable at a temperature of 35 °C only for the chloroplast membranes that had been isolated from the Delisa cultured at 20 °C (but not for the mutant). No differences were observed for the membranes of the plants acclimated at 27 °C (Figure 4F). To conclude, for measurement of the chloroplasts with an incorporated SASL-16 spin label, we can say that the chloroplast membranes of the mutant 522DK that had been acclimated at 5 °C were more ordered (more rigid) than the membranes of the Delisa. Studies using a Langmuir bath led to similar conclusions (author’s unpublished data). The monolayers were built from galactolipids (MGDG, DGDG—the main chloroplast lipid constituents [55]) and also from phospholipids that were isolated from the 522DK mutant and its wild type that had been acclimated at 5 °C. In the case of the mutant, the monolayers were characterised by statistically significantly lower (than Delisa) values of A_lim_. A_lim_ represents the area that is occupied by a single molecule in a completely packed layer, provides information about membrane fluidity and decreases with a decrease in fluidity. In case of monolayers built from MGDG, values of A_lim_ [Å^2^] were 65.6 ± 0.2 and 64.9 ± 0.3, respectively for Delisa and mutant. In case of monolayers built from DGDG values of A_lim_ were 55.2 ± 0.1 and 51.7 ± 0.2, respectively for Delisa and mutant. Usually, greater fluidity of cell membranes is considered as more favourable for a higher frost tolerance, which has been wider discussed in our earlier work [13]. The 522DK mutant, which has a slightly higher frost tolerance than the wild type Delisa [20], is, however, characterized by a greater rigidity of chloroplast membranes than Delisa, as shown in EPR studies. The question remains if/how fact of rigidification of chloroplast membranes is related to the frost tolerance of the mutant. Interestingly, as for the plasma membrane, it is known that the level of the expression of many cold-inducible genes increases two- to three-fold after the rigidification [56]. 

On the other hand, we have to mention that the 522DK mutant that was studied in the present experiment also had a better high-temperature tolerance than the wild-type Delisa [20], although there was no difference in the membrane molecular dynamics between the mutant and the wild type after acclimation at 27 °C (Figure 4C,F). Interestingly, it is also worth noting here that most of the changes in the FA content were observed at 27 °C, but that no differences were observed in the molecular dynamics of the chloroplast membranes of plants that had been acclimated at 27 °C in either the hydrophilic and hydrophobic area. 

**Figure 5:**
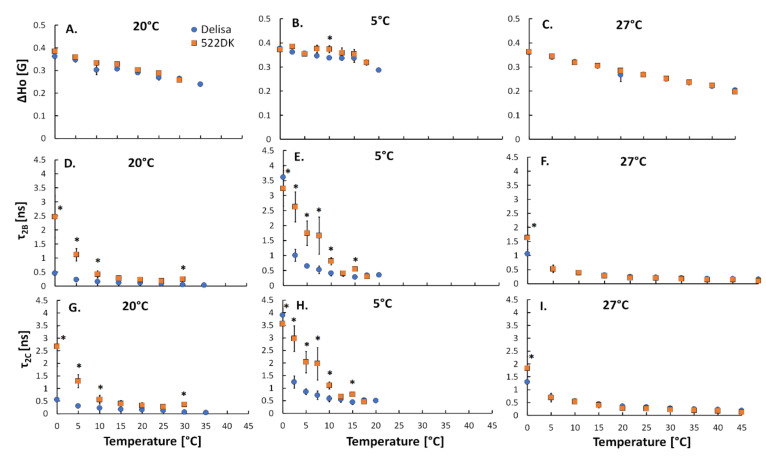
Dependence of parameters ΔH_0_ (**A**–**C**), τ_2B_ (**D**–**F**) and τ_2C_ (**G**–**I**), which were calculated for the chloroplast membranes that were isolated from the barley wild-type Delisa and its mutant 522DK with incorporated SASL-16 on temperature. Plants had been cultivated at 20 °C and acclimated at 5 °C and 27 °C. Average data are given ±SE and significant differences between the wild type and mutant are indicated with an asterisk (*); comparisons were made separately for each EPR measurement temperature.

Analysis of the other three parameters that were calculated from the SASL-16 spectra (ΔH_0_ and rotational correlation times τ_2B_ and τ_2C_) provided additional information about the movement of a spin label and at the same time about the movement of FA in the interior of the membrane. The ΔH_0_ parameter provided information on the oscillation and rotation of a spin label and its rotation along the long axis of the molecule [15]. Lower values of ΔH_0_ indicate a greater freedom of the spin label motion. Our results show that there were generally no differences in the motion of the molecules (ΔH_0_ parameter) of the chloroplast membranes of the Delisa cultivar and its 522DK mutant at the tested temperatures (Figure 5A,B,C). For the other two parameters, τ_2B_ corresponded to the rotation of the spin label molecule along its long axis, while τ_2C_ provided information about the movement of the molecule in the direction perpendicular to the long axis. The rotational correlation times are assumed to reflect the local fluidity of a membrane—lower values indicate a greater motional freedom of the spin labelled FA and thus a higher fluidity of a membrane [14]. 

The values of τ_2B_ and τ_2C_ of 16-SASL in the chloroplast membranes from both Delisa and 522DK depended on the temperature of the measurement (Figure 5D–I). It was observed that within a temperature range between 0 °C and 7.5 °C, the chloroplast membranes of the mutant 522DK, which had been grown at 20 °C and acclimated at 5 °C, was characterised by significantly higher values of τ_2B_ than its wild type (Figure 5D,E). For the plants that had been acclimated at 27 °C, Delisa had a higher value of τ_2B_ than its mutant only around 0 °C (Figure 5F). Moreover, the values of τ_2C_ were noticeably higher for the chloroplast membranes of the 522DK mutant than its wild type, which had been cultivated at 20 °C and acclimated at 5 °C, within a range of measurement temperatures between 0 °C and 10 °C (Figure 5G,H). For the chloroplast membranes of the plants that had been acclimated at 27 °C, there were significant differences between Delisa and the mutant only at a measurement temperature of 0 °C (Figure 5I). When the temperature of the measurement was higher, both parameters had lower values. This decrease in the rate of the molecule rotation along the long axis (τ_2B_) is in line with an increased rigidity of membranes [14].

We can assume that the greater motional freedom had FA included in Delisa’s chloroplast membranes compared to chloroplast membranes of 522DK. Therefore, all of the calculated parameters once again implied that the chloroplast membranes of 522DK at 20 °C and 5 °C were more ordered/less fluid than the chloroplast membranes of the Delisa cultivar. 

The final matter to discuss is what caused the differences in the molecular dynamics of the membranes between the mutant and its wild type. Because the components that are important for membrane structure and properties, such as FA, carotenoids and chlorophylls were comparable in the chloroplasts of the wild type and mutant (Table 1, Table S1, Figure 2), it is likely that the differences in the membrane molecular dynamics might be connected with other components including BR. Among all of the BR found in current study, the concentration of castasterone and homocastasterone was usually at the highest level in the chloroplasts. In the chloroplasts of the cold-treated plants, these BRs were found in amounts of about 1000–6000 pg per g of F.W. The 522DK mutant that had been acclimated at 5 °C had more rigidified chloroplast membranes than the wild type, but also had a similar content of these two BR present in high content (Figure 3A,E). This may suggest that these two BR are not the cause of the differences in the membrane molecular dynamics. The chloroplast content of BR from the other groups such as homodolicholide, dolicholide, and homodolichosterone is a different matter. The cold-acclimated 522DK had a significantly higher content of these BR in the chloroplasts than the wild type (Figure 3D,G,H); however, their content was generally very low (not more than 30 pg per g of F.W.). Thus, it is difficult to say whether they can be responsible for the changes in the molecular dynamics. In turn, it should be remembered that the molecular dynamics of membranes was quite similar in the mutant and the wild type at 20 °C and 27 °C, although there were differences in the BR content. To conclude, the role of BR as membrane stabilisers remains an open question. We cannot totally reject the hypothesis about the role of BR in modifying the dynamics of membranes because there are other studies that suggest this. Li et al. [57] investigated the effect of exogenous BR on the fluidity of the plasma membrane in mango fruits that had been stored at 5 °C. Using the EPR method, the authors found that treatment with BR increased the membrane fluidity in mango fruit. In our earlier studies using the Langmuir bath, we showed that BR are incorporated into the structure of the membrane monolayer from lipids that were isolated from not-hardened and cold-hardened wheat. The changes in the physicochemical parameters of the monolayers such as compressibility were observed [41]. For further investigation of the role of BR as membrane stabilisers studies of EPR on model membranes would be helpful. We can also not exclude the fact that the amount of BR is not crucial here. Perhaps the proportions between each component of membranes including BR as well as sterols, which were not tested here, or some proteins are also important in regulating the chloroplast membrane fluidity. Theoretically, BR can also be located in so-called rafts such as cholesterol in animal cell membranes [58] or phytosterols in plant membranes [59], which affect the membrane properties differently because they are local. Rafts are specialised lipid domains and the role of phytosterols-rich rafts is considered not without merit in maintaining plant membranes in a state of dynamics that is less sensitive to temperature shocks [59]. However, this point of view regarding BR requires further studies. 

## 4. Concluding Remarks

Based on our experiment, the following main findings can be pointed out: 

(1) In the chloroplasts that were isolated from the barley wild-type Delisa and the mutant 522DK, regardless of the plant growth temperature, eight brassinosteroids at different concentrations were identified. Their diverse presence may indicate that they play some role in the functioning of these organelles. 

(2) Mutant 522DK is a BR-deficient mutant that has a lower accumulation of BR compared to the wild type in its leaf tissue [18,20]. However, in the case of the chloroplasts, this regularity has not been proven in present studies. The mutant’s chloroplasts had a higher level of three brassinosteroids: homodolicholide (regardless of the plant growth temperature), dolicholide (plants at 5 °C) and homodolichosterone (plants at 20 °C) than the wild-type Delisa. On the other hand, the content of homocastasterone was lower in the mutant’s chloroplasts (plants at 20 °C), while castasterone was unchanged (regardless of the plant growth temperature). It is likely that the incorporation of BR into the chloroplast membranes is driven by accumulation mechanisms that do not have much in common with their biosynthesis process or the final BR production/concentration in cells. 

(3) Although electron paramagnetic resonance has been used to study the cell membrane properties and the influence of different factors on those properties [60], knowledge about the influence of BR on the fluidity of cell membranes is still rudimentary. In the current work, it was shown that the main components that are important for the chloroplast membrane’s structure/properties, such as FA, carotenoids and chlorophylls, were comparable in the chloroplasts of the wild type and mutant 522DK, but it cannot be unambiguously stated that the BR that were present in the chloroplasts were responsible for the differences in the chloroplast membrane molecular dynamics between the tested genotypes. However, in the hydrophobic (but not hydrophilic) area, the chloroplast membranes of the mutant 522DK that had been acclimated at 5 °C were more ordered (more rigid) than the membranes of the wild-type Delisa. Slight differences between the mutant and wild type were also observed after their growth at 20 °C. There was practically no difference in the membrane dynamics between the mutant and the wild type after acclimation at 27 °C in either the hydrophilic or hydrophobic area. 

## Figures and Tables

**Table 1:** Composition of the fatty acids of the chloroplast membranes isolated from the barley wild-type Delisa and its 522DK mutant cultured at 20 °C, 5 °C, and 27 °C. Any significant differences between Delisa and the mutant (Student’s t-test, *p ≤* 0.05) for each temperature are indicated by different letters. <LOD—below limit of detection.

Fatty Acids [%mol]	Growth Temperature					
	20 °C		5 °C		27 °C	
	Delisa	522DK	Delisa	522DK	Delisa	522DK
10:0	<LOD	0.79	0.47 a	0.50 a	<LOD	0.35
12:0	4.00 a	3.93 a	4.49 a	4.37 a	4.12 b	4.60 a
14:0	0.28 a	0.25 a	0.23 a	0.19 a	0.29 a	0.21 b
16:0	16.54 b	18.76 a	16.16 a	17.41 a	17.20 a	16.11 a
16:1	6.29 a	4.95 a	4.25 a	4.23 a	6.45 a	3.36 a
18:0	3.53 a	3.24 a	3.37 a	3.47 a	2.96 a	2.59 a
18:1 ^Δ9 *cis*^	1.05 b	1.84 a	0.93 b	1.15 a	1.36 a	1.54 a
18:2 ^Δ6 *cis*^	4.24 a	4.32 a	3.05 a	3.01 a	6.85 b	7.64 a
18:3 (3)	63.87 a	61.85 a	66.45 a	65.33 a	60.43 a	63.28 a
20:1	0.19 a	0.06 a	0.34 a	0.34 a	0.34 a	0.31 a
18:3/18:2	15.09 a	14.38 a	22.29 a	21.70 a	8.84 a	8.29 a
U/S	3.11 a	2.72 b	3.01 a	2.86 a	3.08 a	3.20 a

## Data Availability

Data is contained within the article or Supplementary Material.

## Supplementary Materials

The following are available online at https://www.mdpi.com/2218-273X/11/1/27/s1, Figure S1: Sample of the freshly isolated chloroplasts from fresh plant material taken to confirm the purity of the chloroplasts, seen under a Nikon Eclipse E600 microscope (Tokyo, Japan), Figure S2: Dependence of the dynamic orientational order parameter S calculated for the chloroplast membranes (with incorporated SASL-5 (A,B) and SASL-16 (C,D)) that were isolated from the barley wild-type Delisa (A,C) and its mutant 522DK (B,D) that had been cultivated at 20 °C and acclimated at 5 °C and 27 °C on temperature. Average data are given ±SE and significant differences between the temperatures of growth (20 °C, 5 °C, and 27 °C) are indicated with an asterisk (*); comparisons were made separately for each EPR measurement temperature, Table S1: Changes in the content of FA and BR in the barley chloroplasts of the BR-deficient mutant 522DK (mutation *HvDWARF*) compared to the wild-type Delisa (↑ increase compared to the wild type; ↓ decrease compared to the wild type; NC no change compared to the wild type).
