# Supplementary material for: Molecular Dynamics of Chloroplast Membranes Isolated from Wild-Type Barley and a Brassinosteroid-Deficient Mutant Acclimated to Low and High Temperatures
Source: Biomolecules. 2020 Dec 29;11(1):27. doi: 10.3390/biom11010027 (PMC7823496; doi:10.3390/biom11010027)
Supplement: Supplementary file 1 [file biomolecules-11-00027-s001.zip › Table S1..docx]

| **Compounds of chloroplast membranes** | **Growth temperature** | | |
| --- | --- | --- | --- |
|  | **20**°**C** | **5**°**C** | **27**°**C** |
| **FA** |  |  |  |
| 10:0 | ↑ | NC | ↑ |
| 12:0 | NC | NC | ↑ |
| 14:0 | NC | NC | ↓ |
| 16:0 | ↑ | NC | NC |
| 16:1 | NC | NC | NC |
| 18:0 | NC | NC | NC |
| 18:1 ^Δ9^ *^cis^* | ↑ | ↑ | NC |
| 18:2 ^Δ6^ *^cis^* | NC | NC | ↑ |
| 18:3 (3) | NC | NC | NC |
| 20:1 | NC | NC | NC |
| **BR** |  |  |  |
| Homocastasterone | ↓ | NC | NC |
| 28-norcastasterone | ↓ | NC | NC |
| Castasterone | NC | NC | NC |
| Brassinolide | NC | NC | ↑ |
| 24-epibrassinolide | NC | NC | NC |
| Dolicholide | NC | ↑ | ↓ |
| Homodolicholide | ↑ | ↑ | ↑ |
| Homodolichosterone | ↑ | ↑ | ↓ |
| Total BR content | ↓ | NC | NC |

Table S1. Changes in the content of FA and BR in the barley chloroplasts of the BR-deficient mutant 522DK (mutation *HvDWARF*) compared to the wild-type Delisa (↑ increase compared to the wild type; ↓ decrease compared to the wild type; NC no change compared to the wild type).
