# Supplementary figures and images for: Molecular Dynamics of Chloroplast Membranes Isolated from Wild-Type Barley and a Brassinosteroid-Deficient Mutant Acclimated to Low and High Temperatures
Source: Biomolecules. 2020 Dec 29;11(1):27. doi: 10.3390/biom11010027 (PMC7823496; doi:10.3390/biom11010027)

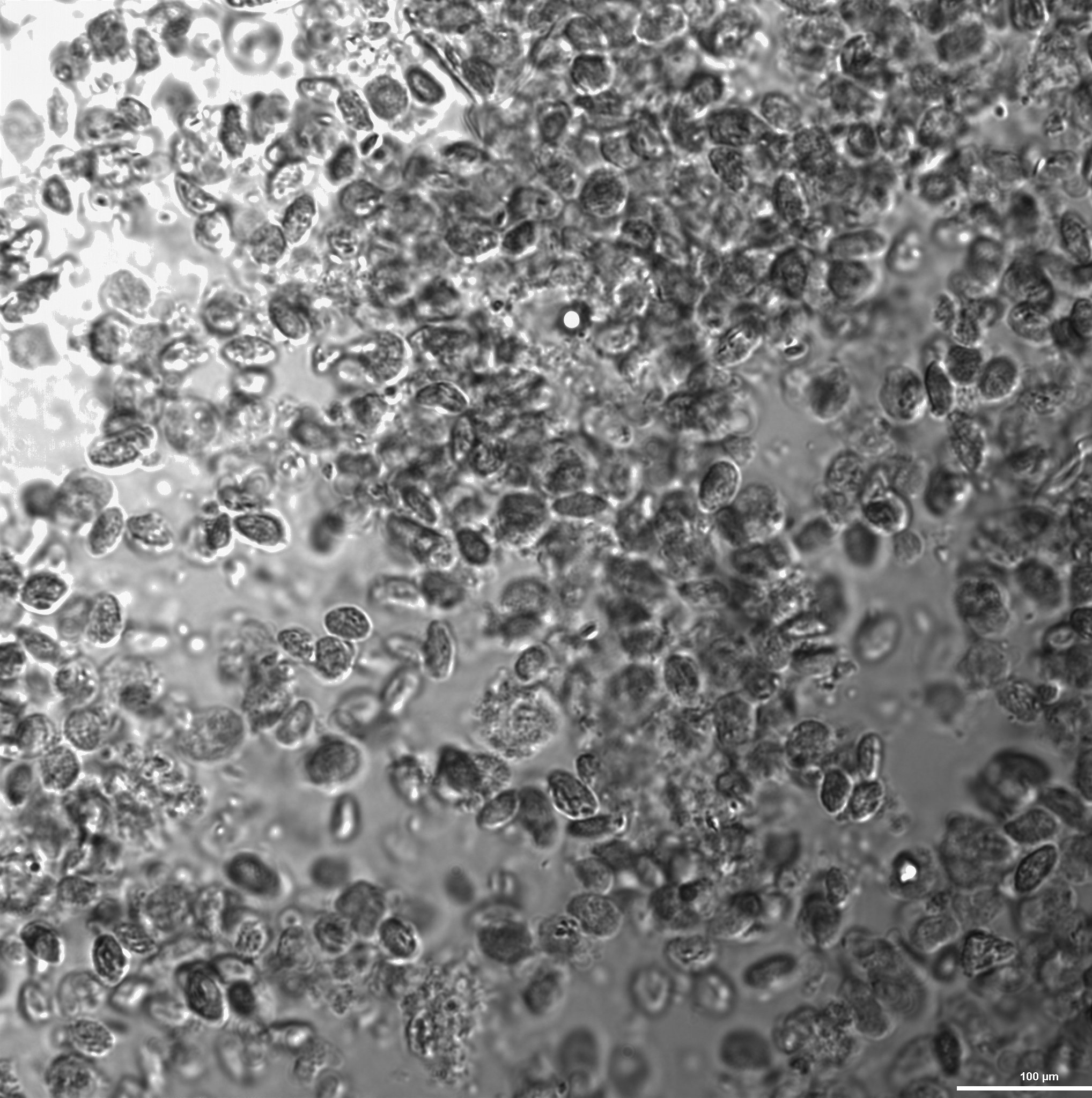

Supplement: Supplementary file 1 [file biomolecules-11-00027-s001.zip › Fig.S1.tif]

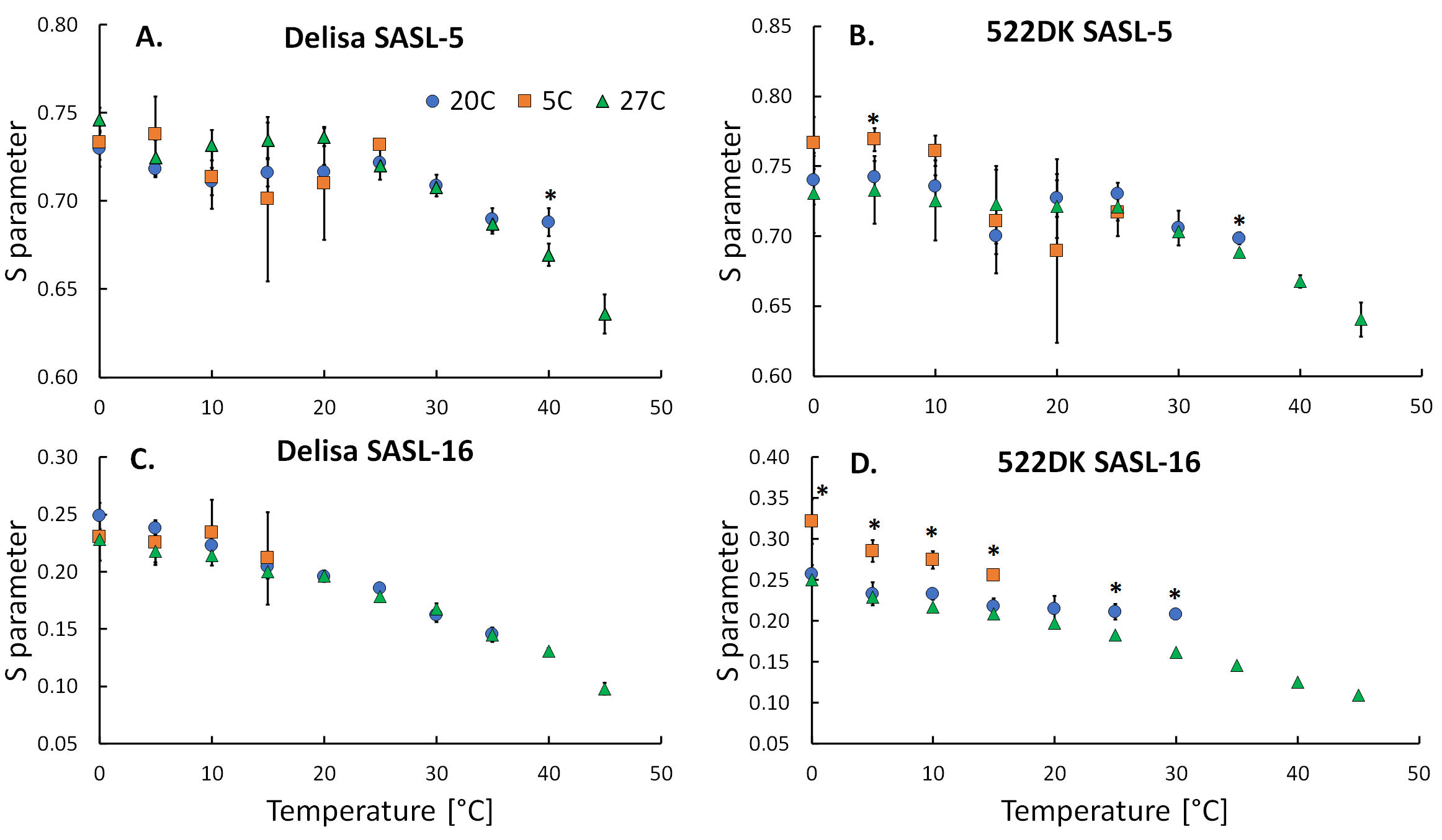

Supplement: Supplementary file 1 [file biomolecules-11-00027-s001.zip › Fig.S2.tif]
